# DEB-TACE with irinotecan *versus* C-TACE for unresectable intrahepatic cholangiocarcinoma: a prospective clinical study

**DOI:** 10.3389/fbioe.2022.1112500

**Published:** 2023-01-12

**Authors:** Junxiao Wang, Yaoqin Xue, Rui Liu, Zhenyu Wen, Zhenhu Ma, Xiang Yang, Lingxiang Yu, Bin Yang, Hui Xie

**Affiliations:** ^1^ Aerospace Medical Center, Aerospace Center Hospital, Beijing, China; ^2^ Senior Department of Oncology, Fifth Medical Center of Chinese PLA General Hospital, Beijing, China; ^3^ Department of Interventional Therapy, Shanxi Province Cancer Hospital, Taiyuan, China; ^4^ Department of Interventional Vascular, Aerospace Center Hospital, Beijing, China; ^5^ Senior Department of Liver Disease, Fifth Medical Center of Chinese PLA General Hospital, Beijing, China

**Keywords:** intrahepatic cholangiocarcinoma, trans-catheter arterial chemoembolisation, irinotecan, drug-eluting beads, efficacy and safety

## Abstract

**Objectives:** DEB-TACE with irinotecan and C-TACE were compared with regards to safety and efficacy for the therapy of intrahepatic cholangiocarcinoma (ICC).

**Methods:** Institutional Review Board approved our trial and we registered it in the Chinese Clinical Trial Registry (ChiCTR1900022856). Forty patients with biopsy-confirmed ICC were randomised to either receive DEB-TACE or C-TACE treatment with 20 patients in each treatment arm. The primary endpoints objective response rate (ORR) and progression free survival (PFS) using the mRECIST to evaluate the tumours. The secondary endpoints were overall survival (OS) and safety. The chi-square was used to analyse the data. The Kaplan-Meier method and Cox analysis were used to evaluate the survival data.

**Results:** ORR (70% in DEB-TACE group vs. 20% in C-TACE, *p* = .001) at 1 month after therapy, ORR (50% vs. 15%, *p* = .018) at 3 months and DCR (70% vs. 30%, *p* = .011) at 6 months, while no difference was found in other groups. (all *p* > .05) The median PFS with DEB-TACE was longer than that with C-TACE (8.0 months vs. 3.0 months) (*p* = .042). Although the median OS was longer with DEB-TACE than with C-TACE (11.5 months vs. 9.0 months), the difference was not statistically significant (*p* = .280). The Cox regression analysis demonstrated that TACE sessions (*p* = .017) and low CA125 levels (*p* = .001) were independent favourable prognostic factors. The most frequent adverse event was elevated transaminase levels (20/20 in DEB-TACE group vs. 15/20 in C-TACE group) (*p* = .047).

**Conclusion:** Our prospective study suggested better ORR and PFS with DEB-TACE with irinotecan as compared to C-TACE with irinotecan in the treatment of unresectable ICC.

## Introduction

Intrahepatic cholangiocarcinoma (ICC) is the relatively common primary hepatic carcinoma excepted for hepatocellular carcinoma. ICC accounts for approximately 10%–20% of newly diagnosed cases of primary liver cancer (Gupta and Dixon, 2017). ICC is an epithelial cell malignancy arising from cholangiocytes within the liver. It has recently attracted attention as its incidence and associated mortality rate have increased globally (Razumilava and Gores, 2014).

Although surgical resection of the tumour such that the margins are histologically negative is the only available cure, this is achievable in less than 30% of patients, and the 5 years survival rate with patients is only 20% (Guglielmi et al., 2009). For patients with unresectable ICC, TACE is an acceptable therapy. However, the median overall survival for these patients is only 12 months and 5 years survival rate less than 5% (Chiang et al., 2020; Massani et al., 2021; Tan et al., 2021).

DEB-TACE is a common palliative therapy and interventional therapy for liver cancer and the sustained release of drugs and the maintenance of drug concentrations within the tumour play important roles. Combination of chemotherapeutic and embolic agents used in TACE are thought to work synergistically to simultaneously limit blood supply to the tumour and increase the local effect of chemotherapeutic agents. Drug-eluting beads TACE (DEB-TACE) tries to provide a steady release of therapeutic drug, hoping to improve overall chemotherapeutic effect, as opposed to conventional TACE (C-TACE) which more rapidly release drug and may overshoot and then undershoot therapeutic drug thresholds (Zhang et al., 2017). CalliSphere is the first bead product completely produced in China and used for DEB-TACE in recent years, and several clinical studies and trials presented satisfying efficacy and safety in DEB-TACE for ICC patients (Luo et al., 2020; Zhou et al., 2020).

Irinotecan-eluting beads TACE has been studies in the setting of colorectal cancer liver metastases, however, there is significantly less data on its use in setting of ICC. Irinotecan is a second-line therapy option for metastatic colon cancers that are insensitive to 5-fluorouracil (Tsujino et al., 2017). As the epithelial cells of the bile ducts are a single layer of columnar epithelial cells that secrete mucus, and the submucosa is surrounded by submucosal glands and smooth muscle cells (Kondo and Fukusato, 2015). It is expected that irinotecan could be a promising therapy for ICC. Numerous studies worldwide have examined the use of irinotecan in the therapy of cholangiocarcinoma such as the study of DEB-TACE with irinotecan vs. doxorubicin as a second line treatment for ICC (Venturini et al., 2016), but DEB-TACE with irinotecan and C-TACE are rarely compared in some clinical trials. Therefore, this prospective randomised trial of 40 patients with ICC aimed to compare the difference of safety and efficacy of DEB-TACE with irinotecan and C-TACE.

## Materials and methods

### Study design

This study was performed at the Fifth Medical Centre of Chinese PLA General Hospital between 2019 and 2021 and was a single-centre, prospective, randomised, controlled trial that aimed to compare the safety and efficacy of DEB-TACE and C-TACE, both with irinotecan, in the treatment of patients with Stage IIIA/B/C ICC, as defined by the TNM classification system.

Informed consent was obtained from every study patient and/or their legal guardian. This study was approved by the Ethical Committee of the Fifth Medical Centre of the Chinese PLA General Hospital. We registered this trial on the Chinese Clinical Trial Registry (ChiCTR1900022856) in 2019.

### This trial of sample size calculation

In previous studies, the ORR of patients who underwent DEB-TACE and C-TACE was 67.6% and 23%, respectively (Savic et al., 2017; Luo et al., 2020). Using a two-sided type I error of .05 and power of 80%, we calculated requiring 40 enrolled participants, 20 in each treatment arm.

### Patients

Our study recruited 101 patients. Inclusion criteria were age >18 years; diagnosis biopsy-confirmed or Chinese liver cancer diagnostic criteria (Zhou et al., 2018) based on CT and MRI; ICC of stage IIIA/B/C according to the TNM classification; ECOG score <2; Child–Pugh class A and B; expected survival at least 6 weeks; adequate liver function and renal function; and at least one measured lesion, according to the mRECIST assessment for ICC(Lencioni, 2013).

Exclusion criteria were severe jaundice and massive ascites; other cachexia and extreme physical weakness; Child–Pugh liver function class C; serious cardiac dysfunction or liver dysfunction; renal failure; mental disorder; active infection, particularly pancreaticobiliary infection; patients who previously underwent other treatments, such as TACE and local ablation; patients with greater than 70% tumour load of the liver and thrombosis of the main portal vein, upper gastrointestinal bleeding within the past 3 months, or HIV infection.

### Randomization and grouping

Our study enrolled in total 40 patients with unresectable ICC. 40 patients were randomly assigned to two group using random numbers table: 20 patients in DEB-TACE group who received DEB-TACE with irinotecan, and 20 patients in C-TACE group who received C-TACE with irinotecan.

### DEB-TACE procedure and therapy

The diameter of CalliSphere Beads (CB; Jiangsu Hengrui Medicine Co., Ltd.) was 100–300 μm used in this study. Beads were mixed with irinotecan (Jiangsu Hengrui Medicine Co., Ltd.) after the initiation of the operation. CB were extracted from a bottle using a 20 mL syringe, and the syringe was placed upright for 2–3 min until the CB settled. The supernatant was then removed. Thereafter, a 10 mL syringe was used to extract saline for injection to dissolve 80 mg irinotecan at a concentration of 20 g/L. The CB were mixed with irinotecan, and the mixture was shaken once every 5 min for 30 min (six times). The drug loading time was 30 min. After irinotecan was adsorbed by the CB, non-ionic contrast agents (Jiangsu Hengrui Medicine Co., Ltd.) were added to the CB mixture at a ratio of 1:1. Finally, the interventional radiologist shaken the mixture and placed in a beaker for 5 min.

A 5-F angiographic catheter (RF catheter; Terumo) was inserted in the celiac trunk and superior mesenteric to detect the arteries feeding the tumors with digital subtraction angiography and the hepatic artery was intubated using the Seldinger technique. A 2.6-F–2.8-F microcatheter (SP microcatheter; Terumo) was used to superselect the tumour-feeding artery. The mixture of CB, irinotecan, and contrast agent was extracted using a syringe and injected into the superselected site through a microcatheter. Embolisation was stopped when the flow rate of the contrast agent became slow or stagnant. Angiography was performed again, the stain lesion disappeared and the operation was ended.

TACE was repeated after 3 months if residual tumours or new lesions were confirmed but it was no longer received TACE according to the following criteria: Child-Pugh class of C, ECOG score >2, international normalised ratio >2.5, liver and kidney function damage, and life expectancy <3 months.

### C-TACE procedure

Similarly, A 5-F angiographic catheter was inserted in the celiac trunk and superior mesenteric to detect the arteries feeding the tumors. 2.6F–2.8F microcatheter was used to superselectively place the tumour feeding arteries. Iodized oil (Lipiodol Ultrafludio; Guerbet, Aulnay-sous-Bois, France) was mixed with irinotecan (the iodized oil is 5–20 mL and the irinotecan is 80 mg) and injected through a microcatheter. Finally, a gelatin sponge was used to block the tumour blood vessels. Angiography was performed again, the stain lesion disappeared and the operation was ended. Similarly, C-TACE was repeated after 3 months.

### Follow-up assessments (trial endpoints)

At baseline, total patients received a physical examination, and their medical history was taken. Assessments of Child–Pugh class, ECOG performance status, and CA125 levels and routine laboratory tests were conducted. Tumour assessments included contrast-enhanced CT (Siemens Sensation Cardiac; Siemens Medical Solutions) and MRI (Magneton Aera; Siemens Medical Solutions). The primary endpoints were tumour response and PFS, and the secondary endpoints were OS and the incidence of adverse events. All patients were assessed using abdominal contrast-enhanced CT or MRI was performed 1 month after TACE and then assess response of treatment every 8–12 weeks. MRI and CT images were evaluated independently in a blinded fashion by two authors with >5 years of experience. Tumour response using the mRECIST was evaluated and it was defined as CR, PR, SD, and PD. The ORR was defined as the population of CR and PR in all patients, and the DCR was defined as the patients with CR, PR and SD. OS was defined as the time from start of TACE therapy to the date of death or loss to follow-up. PFS was defined as the time from therapy initiation to liver tumour progression, progression of lymph node metastasis, or the development of distant metastases. All AEs using the CTCAE v5.0 were recorded and assessed.

### Statistical analysis

The intention to treat analysis was used to evaluate the safety and efficacy in this study. SPSS 26.0 (IBM) and GraphPad Prism 8.0 (GraphPad) were used to analyse data. Data are displayed as counts (%), means ± standard deviations, or medians (25th–75th). Survival curves was generated by using Kaplan-Meier method, and data were compared using a log-rank test or Tarone–Ware test for two-sided *p* values. Cox proportional hazard regression model was used to evaluate the factors influencing prognostic. The ORR and DCR were calculated, and 95% confidence interval (CI) was computed for two therapy group. Chi-square test was used to compare difference. *p* < .05 was considered as statistically significant.

## Results

### Patient baseline characteristics

**FIGURE 1:**
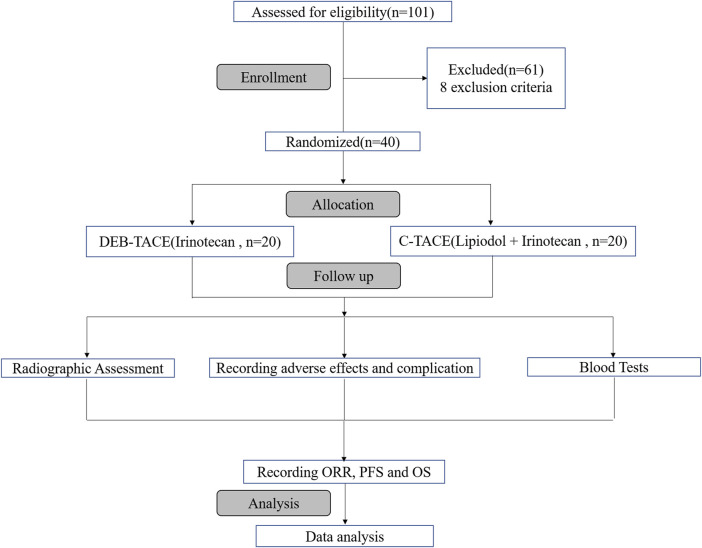
Flowchart of the trial profile.

Of 101 reviewed patients, 40 were randomized for treatment based on inclusion/exclusion criteria. They were randomly allocated to two groups to receive DEB-TACE therapy (irinotecan-eluting beads, *n* = 20) or C-TACE therapy (irinotecan, *n* = 20). The process of selection is summarised (Figure 1).

Overall, we found no significant difference in baseline characteristics between the two randomized groups of 20 patients. In the DEB-TACE and C-TACE groups, 12 (60%) and 10 (50%) patients had periductal infiltration and 15 (75%) and 11 (55%) patients had lymph node involvement, respectively. Tumour size was no statistically significant difference between the two groups. According to the patients’ wishes, 8 patients in D-TACE group and 9 patients in C-TACE group took sorafenib orally after treatment. All patients were not received other local therapy in two groups. The difference in the number of TACE sessions was not statistically significant between the DEB-TACE and C-TACE groups (Table 1).

**TABLE 1:** Baseline characteristics of the study patients.

Parameter	D-TACE (*n* = 20)	C-TACE (*n* = 20)	*p-*value
Age			.490
<60	13 (65)	15 (75)	
>60	7 (35)	5 (25)	
Sex			.082
M	11 (55)	17 (85)	
F	9 (45)	3 (15)	
ECOG performance status			.490
1	13 (65)	15 (75)	
2	7 (35)	5 (25)	
Child-Pugh class			.790
A5	10 (50)	11 (55)	
A6	7 (35)	7 (35)	
B7	2 (10)	2 (10)	
B8	15)	00)	
TNM staging system			.185
ⅢA/B	5 (25)	9 (45)	
ⅢC	15 (75)	11 (55)	
Macroscpic growth patterns			.827
Periductal infiltrate	12 (60)	10 (50)	
Lymph node metastases	15 (75)	11 (55)	
Largest tumor			.240
≤ 5 cm	4 (20)	9 (45)	
5–10 cm	9 (45)	6 (30)	
> 10 cm	7 (35)	5 (25)	
AFP level			
≤ 100 ng/mL	16 (80)	15 (75)	1.000
> 100 ng/mL	4 (20)	5 (25)	
CA19-9 level			.736
≤ 200U/mL	13 (65)	14 (70)	
> 200U/mL	7 (35)	6 (30)	
TACE sessions			1.000
= 1	14 (70)	14 (70)	
> 1	6 (30)	6 (30)	

Note. --- Data were presented as count and percentage. TNM staging system, Tumor Node Metastasis. ECOG, Eastern Cooperative Oncology Group.

### Tumour response

**FIGURE 2:**
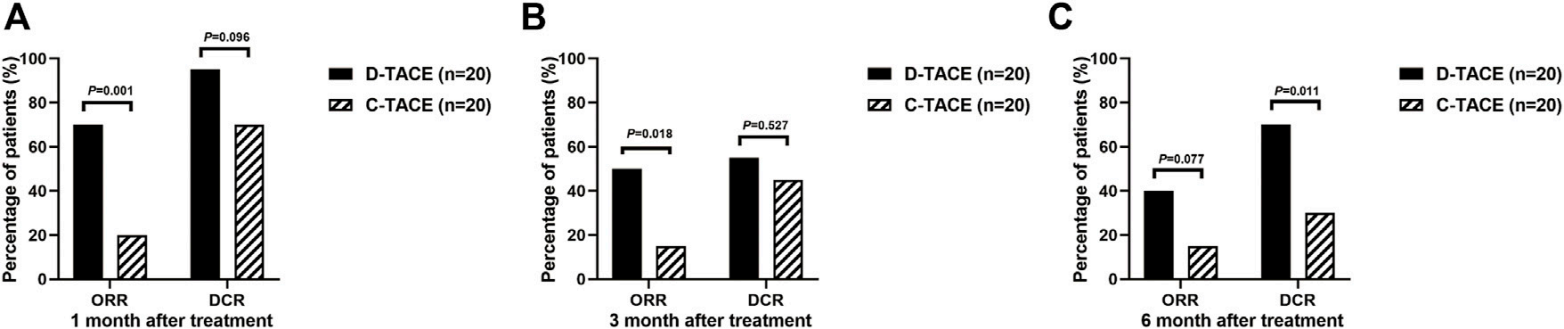
ORR and DCR evaluating by modified Response Evaluation Criteria in Solid Tumors (mRECIST) in D-TACE group and C-TACE group. The comparison of ORR and DCR at 1 month **(A)**, 3 months **(B)**, 6 months **(C)** after therapy between D-TACE group and C-TACE group. CR = complete response, PR = partial response, SD = stable disease, PD = progressive disease. ORR: objective response rate, DCR: disease control rate.

**FIGURE 3:**
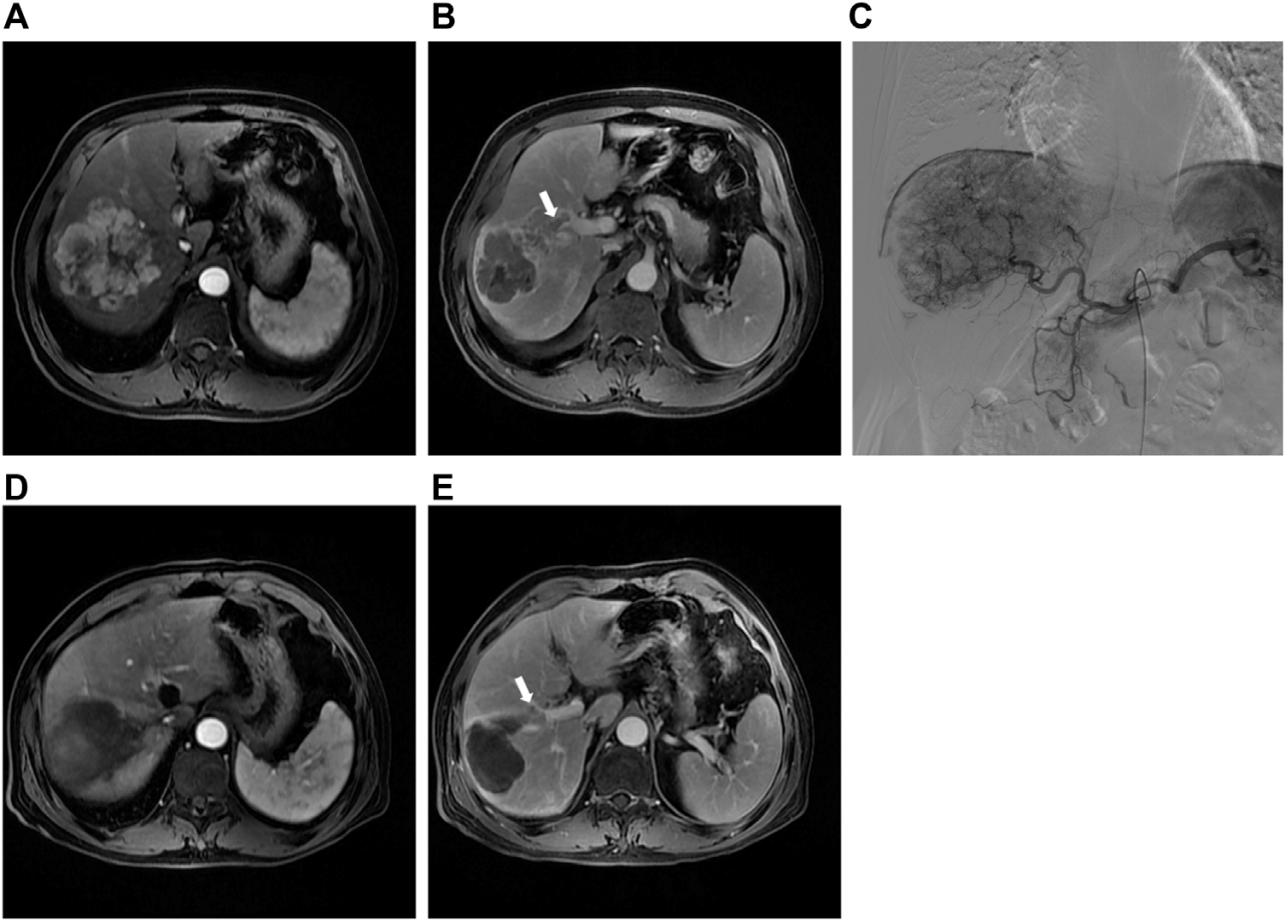
**(A)** Preoperative MRI in the arterial period shows a mass lesion in the right lobe of the liver; **(B)** Preoperative MRI in the delay period shows tumor thrombus of right anterior portal vein; **(C)** The intraoperative angiography showed the staining shadow of the mass tumor in the right lobe of the liver. Two drug-eluting beads (300–500 μm, 80 mL irinotecan) and two embolization beads (300–500 μm) were used to embolization the tumor vessels; **(D)** Abdominal MRI reexamination 3 months after DEB-TACE revealed complete necrosis of the lesion; **(E)** The tumor thrombus of portal vein was significantly smaller than before.

The primary endpoint was ORR in 40 study patients assessed by experienced radiologists using mRECIST criteria (Figure 2). The ORR at 1 month after therapy was 70% in the DEB-TACE group (14 of 20), but in the C-TACE group, the ORR at 1 month was only 20% (2 of 20). Between the two groups, the ORR was statistically significant difference (*p* = .001). DCR (95% in DEB-TACE group vs. 70% in C-TACE group, *p* = .096) at 1 month (Figure 2A). At 3 months ORR (50% vs. 15%, *p* = .018) and DCR (55% vs. 45%, *p* = .527), respectively (Figure 2B). The ORR (40% vs. 20%, *p* = .168) and DCR (70% vs. 30%, *p* = .011) at 6 months after therapy (Figure 2C). Waterfall plot image shows tumor response of 40 patients at 3 months after therapy (Figure 3). CR and PR was observed in 3 of 20 (10%) patients and 7 of 20 (40%) patients in DEB-TACE group. CR and PR was observed in 0 of 20 (0%) and 3 of 20 (15%) patients in C-TACE group at 3 months, respectively. Tumour response of one patient in the DEB-TACE group was showed (Figure 3).

### Survival and disease progression

**FIGURE 4:**
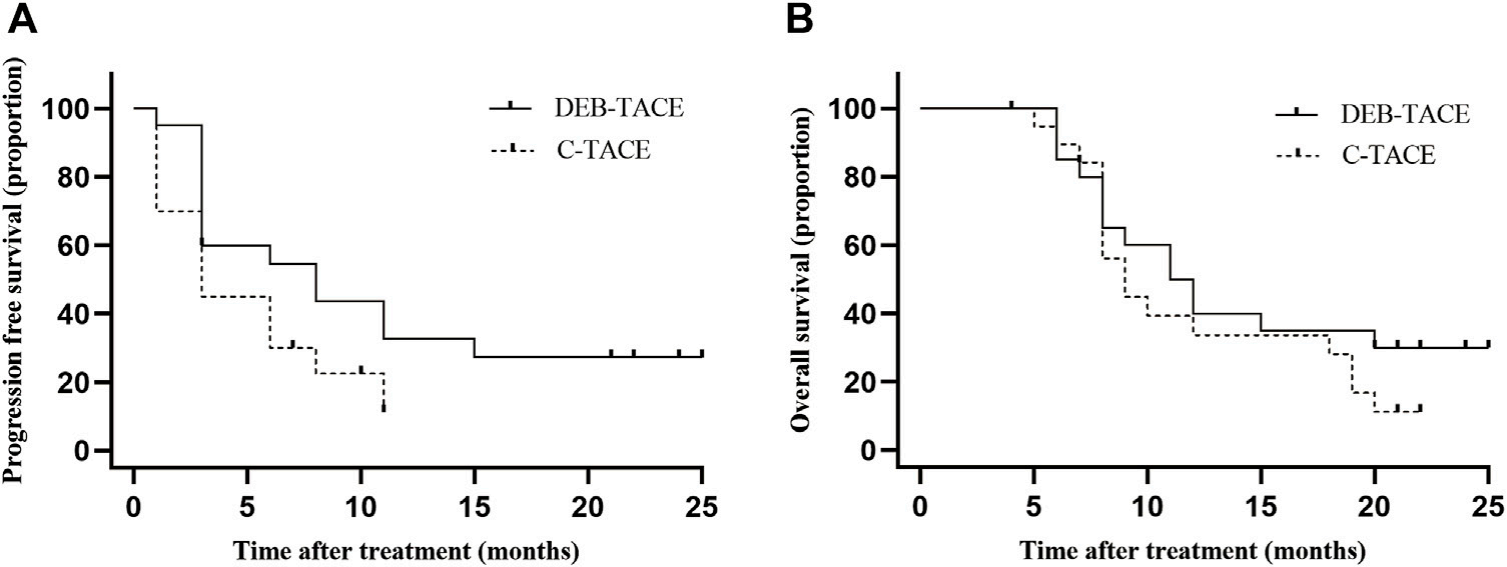
Kaplan-Meier curves shows all participants’ PFS and OS. **(A)** median PFS was 8.0 months (95% CI: 1.2, 14.8) in the DEB-TACE group and 3.0 months (95% CI: 1.6, 4.4) in the C-TACE group (hazard ratio, .5; 95% CI: .24, .94 *p* = .042); **(B)** median OS was 11.5 months (95% CI: 7.7, 14.3) in the DEB-TACE group and 9.0 months (95% CI: 7.0, 11.0) in the C-TACE group (hazard ratio, .78; 95% CI: .38, 1.60 *p* = .280).

In the DEB-TACE group, the median PFS was 8.0 months (95% CI: 1.2, 14.8) and 3.0 months (95% CI: 1.6, 4.4) in the C-TACE group [hazard ratio (HR), .5; 95% CI: .24, .94, *p* = .042] (Figure 4A).

In the DEB-TACE group, the median OS was 11.5 months (95% CI: 7.7, 14.3) and 9.0 months (95% CI: 7.0, 11.0) in the C-TACE group (HR, .78; 95% CI: .38, 1.60, *p* = .280). In the DEB-TACE group, the 1-year survival rate was 50% and was 35% in the C-TACE group (Figure 4B).

### Factors influencing OS of patients with unresectable ICC

**FIGURE 5:**
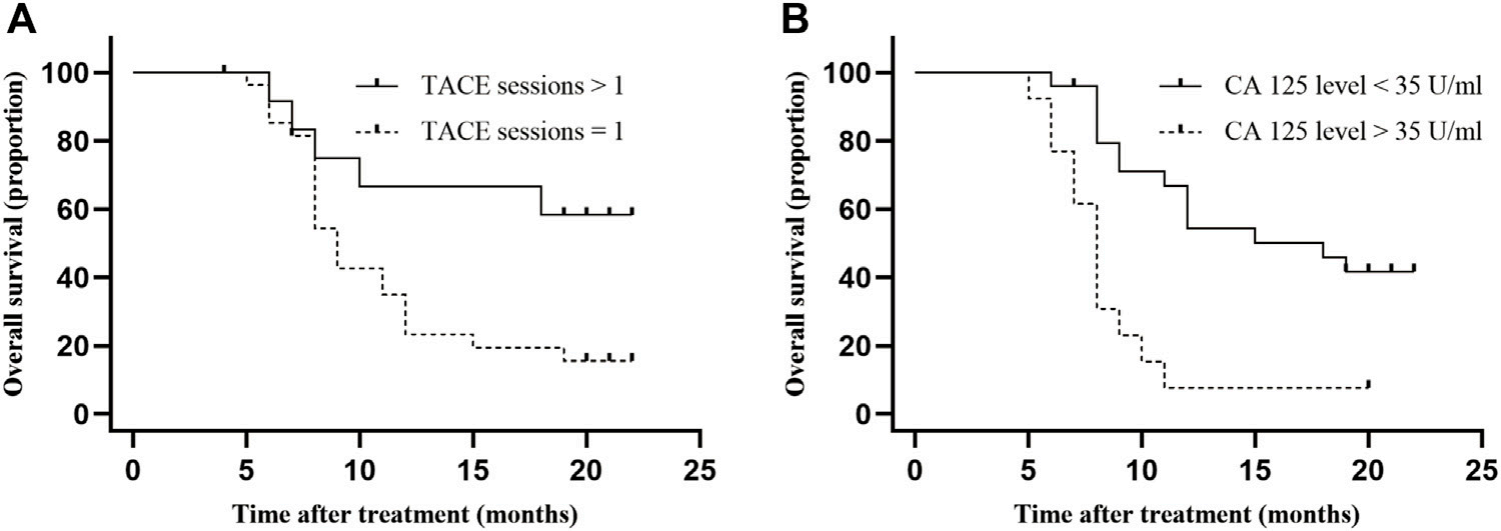
Comparison of OS in subgroup analysis. Independent predictive factors of OS were selected in subgroup analysis to further evaluate their influence on patients’ survival. **(A)** Median OS was 19.5 months in TACE sessions >1 group and 9.0 months (95% CI: 5.9–10.1) in TACE sessions = 1 group, *p*-value = .017; **(B)** Median OS was 18.0 months (95% CI: 5.8–12.2) in low CA 125 level group and 8.0 months (95% CI: .0–25.9) in high CA 125 level group, *p*-value = .001.

Cox analysis was used to assess the information of 40 patients with unresectable ICC treated with TACE (Table 2). The TACE sessions were performed with a univariate regression analysis showed (HR = 2.870, *p* = .036) correlated with longer OS, while High CA125 levels (HR = .265, *p* = .001) were related with shorter OS. A multivariate regression analysis demonstrated that TACE sessions (HR = 3.412, *p* = .017) independently indicated longer OS. In contrast, CA125 levels (HR = .219, *p* = .001) independently indicated shorter OS. We performed the further subgroup analysis to assess the differences in OS between patients with different subgroup (Figure 5). The results demonstrated that TACE sessions (*p* = .020) correlated with longer OS (Figures 5A, B). The median OS was 19.5 months in DEB-TACE group and 9.0 months in C-TACE group with the TACE sessions subgroup analysis. However, CA125 levels related with shorter OS (*p* = .001) (Figure 5B) with a median OS of 18.0 months in the low CA125 levels group and 8.0 months in the high CA125 levels group.

**TABLE 2:** Cox’s proportional hazards regression model analysis of factors affecting OS.

Variable	Univariate analysis			Multivariate analysis		
	HR	95% CI	*P*	HR	95% CI	*P*
Gender (Male/Female)	2.123	.854–5.279	.105	2.123	.854–5.279	.105
Age (≤60/>60)	1.617	.681–3.836	.276			
BMI level (≤23.9/>23.9)	1.707	.794–3.670	.171			
Family history of HCC (Yes/No)	.905	.271–3.024	.871			
History of smoking (Yes/No)	.992	.466–2.111	.983			
History of alcohol (Yes/No)	.990	.465–2.109	.979			
History of diabetes (Yes/No)	3.203	.756–13.578	.114			
TACE sessions (= 1/> 1)	2.870	1.073–7.678	.036	3.412	1.240–9.389	.017
Tumor size (≤5 cm/> 5 cm)	.895	.418–1.914	.774			
Periductal infiltrate (Yes/No)	1.066	.501–2.270	.868			
Lymph node metastases (Yes/No)	.882	.385–2.022	.767			
AFP level (≤10 ng/mL/> 10 ng/mL)	.849	.393–1.830	.675			
CEA level (≤3.4 ng/mL/> 3.4 ng/mL)	.488	.220–1.080	.077			
CA 19–9 level (≤39 U/mL/> 39 U/mL)	.512	.215–1.218	.130			
CA 125 level (≤35 U/mL/> 35 U/mL)	.265	.117–.599	.001	.219	.093–.517	.001
CA 72–4 level (≤8.2 U/mL/> 8.2 U/mL)	.993	.398–2.477	.988			
ECOG performance status (1/2)	1.083	.486–2.413	.845			
TNM staging system (ⅢA/B and ⅢC)	1.080	.472–2.472	.856			

Note. --- Data were presented as HR (hazards ratio), 95% CI (confidence interval) and *p*-value. Prognostic factors affecting OS (overall survival) were determined by univariate and multivariate Cox’s proportional hazards regression model analyses. *p*-value <.05 was statistically significant.

### Safety analysis

We analysed all patients’ data about safety. Most complications are grade Ⅰ-Ⅱ and only one complication is Ⅲ-Ⅳ. The most common TACE-related adverse events were fever (DEB-TACE group, 10/20 [50%] vs. C-TACE group, 11/20 [55%], *p* = .752); abdominal pain (DEB-TACE group, 5/20 [25%] vs. C-TACE group, 8/20 [40%], *p* = .311); hyperbilirubinemia (DEB-TACE group, 10/20 [50%] vs. C-TACE group, 8/20 [40%], *p* = .525); and elevation of transaminase levels (DEB-TACE group, 20/20 [100%] vs. C-TACE group, 15/20 [75%], *p* = .047) (Table 3). Only one patient developed liver abscess after DEB-TACE therapy, which was relieved after percutaneous drainage.

**TABLE 3:** Common toxic effects encountered after treatment.

Toxic effect	D-TACE (*n* = 20)	C-TACE (*n* = 20)	*p-*value	Grade
Nausea	2 (10)	5 (25)	.405	Ⅰ-Ⅱ
Vomiting	15)	15)	1.000	Ⅰ-Ⅱ
Fever	10 (50)	11 (55)	.752	Ⅰ-Ⅱ
Abdominal pain	5 (25)	8 (40)	.311	Ⅰ-Ⅱ
Fatigue	00)	15)	1.000	Ⅰ-Ⅱ
Hypertension	00)	15)	1.000	Ⅰ-Ⅱ
Hyperbilirubinemia	10 (50)	8 (40)	.525	Ⅰ-Ⅱ
Elevation of transaminase	20 (100)	15 (75)	.047	Ⅰ-Ⅱ
Hepatic abscess	15)	00)	1.000	Ⅲ-Ⅳ

Note. --- data were presented as count and percentage. The description was based on 40 DEB-TACE, records.

## Discussion

This study is a prospective clinical trial comparing safety and efficacy of DEB-TACE with irinotecan and C-TACE with irinotecan in the setting of unresectable ICC.

The 3 months ORR in DEB-TACE group was 50% in our study, which was higher than that in patients who underwent C-TACE. However, the ORR in the DEB-TACE group was lower than that in previously reported studies (Marquardt et al., 2019; Zhou et al., 2020). There may be several reasons for this. First, the patients included in this study typically had a poor prognosis as most cases were diagnosed at Ⅲ stage. Second, the sample size was relatively small. Another phase II trial in unresectable cholangiocarcinoma showed that the ORR was only 39.3% (Sohal et al., 2013). Conversely, median PFS (8.0 months, 95% CI: 1.2, 14.8) in the DEB-TACE group of this study was higher than those in previous studies (Zhen et al., 2018; Zhou et al., 2020). Median OS (11.5, 95% CI: 7.7, 14.3) in the DEB-TACE group was similar to those found in previous studies (Kim et al., 2008; Zhou et al., 2020) but was longer than that of the C-TACE group in this study. Several studies of second-line chemotherapy for advanced biliary tract cancer showed that median OS was less than 8 months, and hepatic arterial infusion chemotherapy for advanced cholangiocarcinoma showed median OS was 13.2 months (Chen et al., 2016; Lamarca et al., 2021; Huang et al., 2022). The OS of these studies are similar to ours (median OS in DEB-TACE group: 11.5 months). The multivariate regression analysis demonstrated that TACE sessions, and CA125 levels predicted OS in unresectable ICC patients. Further subgroup analysis showed that TACE sessions (*p* = .020) and CA125 levels <35 U/mL (*p* = .001) were related with longer OS. Previous studies have also demonstrated that TACE sessions, and CA125 levels were factors influencing prognosis (Higashi et al., 2012; Zhou et al., 2020). However, prognostic factors such as tumour burden and lymph node metastasis were not statistically significant in this study, though this may be related to the size of the study sample (Bagante et al., 2019; Asaoka et al., 2020). The median survival of approximately 10 months demonstrated the advantage of TACE sessions in patients with unresectable ICC. CA125 levels may be a factor influencing prognostic in these patients; but this needs to be confirmed by prospective studies of bigger sample size.

Regarding safety, no therapy-related deaths occurred in our study, and only 2.5% (1 of 40) of the patients developed liver abscesses after DEB-TACE therapy, which is not a common occurrence. In a previous study, the most common side effects following TACE were abdominal pain, fever, and elevated transaminase levels (Aliberti, et al., 2008; Carter and Martin Ii, 2009; Schicho et al., 2017). The incidence of side effects is relatively low, and typically no major adverse events occur (Poggi et al., 2008). Similarly, in our study, there were few side effects but almost all patients had mild fever, hyperbilirubinemia, and elevated transaminase levels. All patients in the DEB-TACE group and 15 patients in the C-TACE group showed elevated transaminase levels. Five patients who did not show elevated transaminase levels were taking medications such as magnesium isoglycyrrhizinate. The degree of embolisation, tumour size, and liver function may affect transaminase levels, and this should be addressed in future studies. The incidence of side effects between two treatment groups was similar and most of side effects are grade Ⅰ-Ⅱ, thus, we speculated that DEB-TACE And C-TACE with irinotecan have comparable safety profiles.

TACE is a palliative approach and possible locoregional therapy for liver cancer. It has been demonstrated to be safe and effective in inducing tumour response in unresectable ICC(Savic et al., 2017). DEB-TACE combines the embolisation and drug-eluting properties of beads resulting in selective tumour targeting. In this study, we selected CB beads with diameters of 100–300 μm based on the following reasons. First, smaller beads can behave more aggressively and embolise distally, resulting in a higher concentration of the drug in the tumoral vascular network (Pillai et al., 1991; Yamamoto et al., 2006; Lee et al., 2008). Then, some clinical studies have shown that using 100–300 μm beads may improve efficacy and safety of TACE. Most studies of efficacy and safety of DEB-TACE in ICC using 100–300 μm beads to treat ICC patients (Martin et al., 2022; Yang, et al., 2022). In a retrospective, single institution study conducted from 2010 to 2012, 61 participants were treated with TACE performed with 100–300 μm or 300–500 μm DEBs. The CR in the group for whom 100–300 μm beads were used was higher than that of the group for whom 300–500 μm beads were used (59% and 36%, respectively). In addition, the rates of toxicity were lower when smaller beads were used (Padia et al., 2013). The incidence of postembolisation syndrome and fatigue after therapy were 8% and 36% in the 100–300 μm group and 40% and 70% in the 300–500 μm group, respectively.

Our study also had some limitations. First of all, the sample size was relatively small in this study. Secondly, there was no significant improvement in ORR and OS in our study compared to those in previous studies, because most patients had unresectable ICC. Although therapy modalities for ICC are rapidly progressing, effective therapy modalities for unresectable ICC are still lacking (Colyn et al., 2020; Goyal et al., 2020; Tsilimigras et al., 2020; Meadows and Francis, 2021). Finally, even if imaging data were evaluated by experienced radiologists, it was difficult to use the mRECIST criteria to evaluate the efficacy of TACE in ICC because of irregular necrosis.

In general, the DEB-TACE group showed improvements in ORR and DCR and nearly 5 months in median PFS when compared with the C-TACE group, but the efficacy of DEB-TACE was not satisfied. Consequently, it is crucial to explore the efficacy of combination of loco-regional therapy, immunotherapy, and targeted therapy in patients with unresectable ICC. Meanwhile, the use of enzyme-activatable nanosystem and nanoscale framework might provide more options for the treatment strategy of ICC(Yu et al., 2021; Qiu et al., 2022). We will aim to confirm the value of comprehensive therapy combinations for patients with unresectable ICC in the future.

## Conclusion

(1) The objective response rate (ORR) and progression free survival (PFS) were higher in the DEB-TACE group than in the C-TACE group (ORR 70% vs. 20% at 1 month and mPFS 8.0 months vs. 3 months, respectively).
(2) The incidence of side effects was relatively low, and no major adverse events occurred in either the DEB-TACE or C-TACE group.
(3) TACE sessions and low CA125 levels were independent favourable prognostic factors.

## Data Availability

The raw data supporting the conclusion of this article will be made available by the authors, without undue reservation.

## Abbreviations

AEs, adverse events; CB, CalliSphere Beads; CI, confidence interval; CR, complete response; C-TACE, conventional TACE; CTCAE v5.0, five grade Common Therapy Evaluation Program’s Common Terminology Criteria for Adverse Events Version 5.0; DCR, disease control rate; DEB-TACE, drug-eluting beads TACE; ECOG, Eastern Cooperative Oncology Group; HR, hazards ratio; ICC, Intrahepatic cholangiocarcinoma; mRECIST, modified Response Evaluation Criteria in Solid Tumors; ORR, objective response rate; OS, overall survival; PFS, progression free survival; PR, partial response; SD, stable disease.
